# Quantification of the Resveratrol Analogs *trans*-2,3-Dimethoxy-stilbene and *trans*-3,4-Dimethoxystilbene in Rat Plasma: Application to Pre-Clinical Pharmacokinetic Studies

**DOI:** 10.3390/molecules19079577

**Published:** 2014-07-07

**Authors:** Shermain Yali Ng, Nunzio Cardullo, Samuel Chao Ming Yeo, Carmela Spatafora, Corrado Tringali, Pei-Shi Ong, Hai-Shu Lin

**Affiliations:** 1 Department of Pharmacy, Faculty of Science, National University of Singapore, 10 Kent Ridge Crescent, Singapore 119260, Singapore; E-Mails: ng.yali.shermain@nus.edu.sg (S.Y.N.); yeo.cms@nus.edu.sg (S.C.M.Y.); phaops@nus.edu.sg (P.-S.O.); 2 Dipartimento di Scienze Chimiche, Università di Catania, Viale A. Doria 6, I-95125 Catania, Italy; E-Mails: nunzio.cardullo@hotmail.it (N.C.); cspatafo@unict.it (C.S.); ctringali@unict.it (C.T.)

**Keywords:** resveratrol, *trans*-2,3-dimethoxystilbene, *trans*-3,4-dimethoxystilbene, HPLC, pharmacokinetics

## Abstract

*trans*-2,3-Dimethoxystilbene (2,3-DMS) and *trans*-3,4-dimethoxystilbene (3,4-DMS) are two synthetic resveratrol (*trans*-3,5,4'-trihydroxystilbene) analogs. In this study, a simple HPLC method was developed and validated to determine 2,3-DMS and 3,4-DMS in rat plasma. Chromatographic separation was obtained with a reversed-phase HPLC column through a 12.5-min gradient delivery of a mixture of acetonitrile and water at the flow rate of 1.5 mL/min at 50 °C. The lower limit of quantification was 10 ng/mL. After successful validation, the pharmacokinetic profiles of 2,3-DMS and 3,4-DMS were subsequently studied in Sprague-Dawley rats. Upon single intravenous administration (4 mg/kg), 2,3-DMS had a medium volume of distribution of the central compartment (*V_c_* = 2.71 ± 0.51 L/kg), quite rapid clearance (*Cl* = 52.0 ± 7.0 mL/min/kg), moderate mean transit time (*MTT_0→last_* = 131.0 ± 4.5 min) but a fairly long terminal elimination half-life (*t_1/2_*_λ*Z*_ = 288.9 ± 92.9 min). Interestingly, 3,4-DMS displayed a pharmacokinetic profile apparently distinct from 2,3-DMS and it had more extensive distribution (*V_c_* = 5.58 ± 1.73 L/kg), faster clearance (*Cl* = 143.4 ± 40.5 mL/min/kg) and shorter residence (*MTT_0→last_* = 61.4 ± 27.1 min). Following single oral administration (10 mg/kg), 2,3-DMS had low and erratic plasma exposure (*C_max_* = 37.5 ± 23.7 ng/mL) and poor oral bioavailability (2.22% ± 2.13%) while the oral bioavailability of 3,4-DMS was even poorer than 2,3-DMS. Clearly, the location of the methoxy groups had a significant impact on the pharmacokinetics of resveratrol analogs. This study provided useful information for the design of resveratrol derivatives in future study.

## 1. Introduction

**Figure 1:**
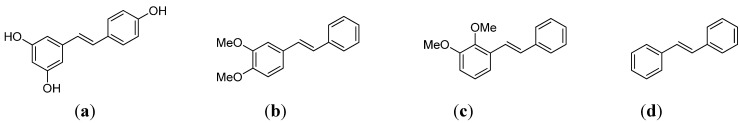
The chemical structures of resveratrol and some of its analogs: (**a**) resveratrol; (**b**) 3,4-DMS; (**c**) 2,3-DMS; (**d**) *trans*-stilbene.

Resveratrol (*trans*-3,5,4'-trihydroxystilbene, Figure 1a) is a dietary phytoalexin that has attracted substantial interest in biomedical research during the last two decades [1]. Its health-promoting activities, including anti-ageing, anti-cancer, anti-diabetic, anti-inflammatory, anti-obesity, anti-oxidation, cardioprotective and neuroprotective properties have been extensively investigated [1]. The interest in resveratrol has also been extended to its naturally occurring and/or synthetic analogs and the beneficial pharmacological activities of these analogs are being identified [2,3,4].

*trans*-3,4-Dimethoxystilbene (3,4-DMS, Figure 1b) is a synthetic resveratrol analog that has exhibited promising anti-angiogenic activities in various pre-clinical models [5]. It effectively inhibited endothelial cell proliferation, migration, tube formation, and endogenous neovascularization [5]. The anti-angiogenic effects of 3,4-DMS might be mediated through the induction of endothelial cell apoptosis [5]. Moreover, it has been reported as an inhibitor of NF-κB activation, an important signaling pathway in inflammation [6]. Since 3,4-DMS has emerged as a lead compound for the development of a new class of drugs targeting angiogenesis-related diseases [5], it is of great interest to explore its pharmacokinetic profiles.

Previous literatures have suggested that the location of methoxy groups had a significant impact on the biological activities [7] and oral pharmacokinetic profiles of methoxystilbenes [8,9,10,11]. To further elucidate the structure-pharmacokinetic relationships, it is also of interest to compare the pharmacokinetic profile of 3,4-DMS with a similar analog. As the only difference between 3,4-DMS and *trans*-2,3-dimethoxystilbene (2,3-DMS) is the location of the second methoxy group, 2,3-DMS appears to be an appropriate candidate for such a comparison.

In this study, a simple HPLC method was developed and validated for the quantification of 2,3-DMS and 3,4-DMS in rat plasma. The pharmacokinetic profiles of 2,3-DMS and 3,4-DMS were subsequently assessed in Sprague-Dawley rats. The information obtained in this study can be used as reference for the design of resveratrol derivatives in future study.

## 2. Results and Discussion

### 2.1. HPLC Method Validation

**Figure 2:**
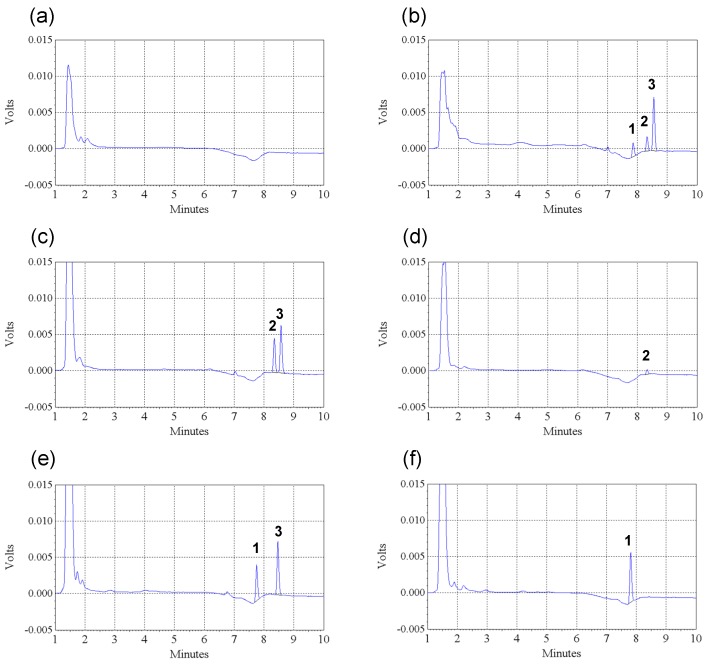
Typical chromatograms (UV absorbance, λ = 310 nm) of (**a**) a pre-dosing plasma sample; (**b**) a blank plasma sample spiked with 3,4-DMS (100 ng/mL), 2,3-DMS (100 ng/mL) and *trans*-stilbene (internal standard, IS) (300 ng/mL); (**c**) a plasma sample taken from a rat at 60 min after receiving an intravenous dose of 2,3-DMS (4 mg/kg), with IS; (**d**) a plasma sample taken from a rat at 15 min after receiving an oral dose of 2,3-DMS (10 mg/kg), without IS; (**e**) a plasma sample taken from a rat at 30 min after receiving an intravenous dose of 3,4-DMS (4 mg/kg), with IS; (**f**) a plasma sample taken from a rat at 15 min after receiving an intravenous dose of 3,4-DMS (4 mg/kg), without IS. Peak 1: 3,4-DMS, peak 2: 2,3-DMS, peak 3: *trans*-stilbene (IS).

The selectivity of this HPLC-UV method for quantification of 2,3-DMS and 3,4-DMS in rat plasma was documented. Under our chromatographic conditions, 3,4-DMS, 2,3-DMS and *trans*-stilbene (IS) elutes at about 7.9 min, 8.3 min and 8.5 min respectively (Figure 2b). No notable interference peak in the chromatograms acquired from either blank plasma samples (*n* = 6) or pre-dosing plasma samples (*n* = 22) was observed (a typical chromatogram of a pre-dosing sample is shown in Figure 2a). Moreover, no notable metabolite or interference peaks were observed in the chromatograms obtained with post-dosing samples (Figure 2c–f).

The lower limit of quantification (LLOQ) of 2,3-DMS and 3,4-DMS, an indicator of the sensitivity of the assay was 10 ng/mL. The calibration curves were all linear with regression correlation coefficients *R^2^* > 0.995. The accuracy and precision of this HPLC-UV method was confirmed. The intra-day and inter-day accuracy and precision of the quality control samples are shown (Table 1 and Table 2). The stability profiles of 2,3-DMS and 3,4-DMS were investigated. 2,3-DMS and 3,4-DMS appeared to be quite stable under the tested conditions (Table 3).

**Table 1:** Intra-day analytical accuracy and precision of 2,3-DMS and 3,4-DMS in rat plasma ^a^.

Amount Spiked (ng/mL)	2,3-DMS			3,4-DMS		
	Amount Measured (ng/mL)	Precision (RSD, %)	Bias Range (%)	Amount Measured (ng/mL)	Precision (RSD, %)	Bias Range (%)
25.0	24.2 ± 1.6	6.3	−3.4 ~ +11.8	24.6 ± 1.0	4.2	−3.9 ~ +7.2
400.0	398.2 ± 3.7	0.9	−0.4 ~ +1.9	409.3 ± 5.1	1.3	−4.0 ~ +0.6
1400.0	1378.0 ± 15.3	1.1	0.8 ~ +3.4	1398.0 ± 21.5	1.5	−0.8 ~ +2.8

^a^ *n* = 5.

**Table 2:** Inter-day analytical accuracy and precision of 2,3-DMS and 3,4-DMS in rat plasma ^a^.

Amount Spiked (ng/mL)	2,3-DMS			3,4-DMS		
	Amount Measured (ng/mL)	Precision (RSD, %)	Bias Range (%)	Amount Measured (ng/mL)	Precision (RSD, %)	Bias Range (%)
25.0	25.2 ± 2.1	8.3	−9.3 ~ +13.6	25.6 ± 2.0	7.7	−13.1 ~ +7.2
400.0	382.4 ± 20.2	5.3	−0.8 ~ +13.1	392.2 ± 19.2	4.8	−3.4 ~ +9.8
1400.0	1340.0 ± 54.6	4.1	0.1 ~ +11.2	1392.0 ± 81.0	5.8	−11.1 ~ +7.5

^a^ *n* = 5.

**Table 3:** Stability Profiles of 2,3-DMS and 3,4-DMS ^a^.

	2,3-DMS				3,4-DMS					
	Stability (% Remained)				Stability (% Remained)					
Stock solution stored at 24 °C for 8 days	98.1 ± 2.1				96.7 ± 2.7					
	Spiked Concentration (ng/mL)				Spiked Concentration (ng/mL)					
	25	400		1400	25			400	1400	
Plasma samples stored at 4 °C for 6 h	94.9 ± 4.4	95.5 ± 1.1		95.4 ± 0.6	104.3 ± 6.6			94.7 ± 0.7	96.2 ± 0.4	
Post-preparative samples stored at 24 °C for 24 h	97.2 ± 2.5	100.1 ± 0.5		100.6 ± 0.7	98.1 ± 5.7			98.1 ± 1.8	100.1 ± 0.6	
Plasma samples after three freeze-thaw cycles	102.3 ± 4.5	93.2 ± 1.2		91.8 ± 2.6	106.1 ± 5.2			93.4 ± 0.7	92.4 ± 2.4	
Plasma samples stored at −80 °C for 10 days	106.9 ± 6.6	96.6 ± 4.7		93.5 ± 4.1	91.0 ±13.8			93.6 ± 4.6	93.0 ± 3.7	

^a^ Results were presented as Mean ± SD (*n* = 5).

A simple and sensitive HPLC-UV method was developed and validated for the quantification of 2,3-DMS and 3,4-DMS in rat plasma. To our knowledge, this is the first validated assay for the quantification of 2,3-DMS and 3,4-DMS in a biological matrix.

### 2.2. Metabolic Stability in Rat Liver Microsome

**Figure 3:**
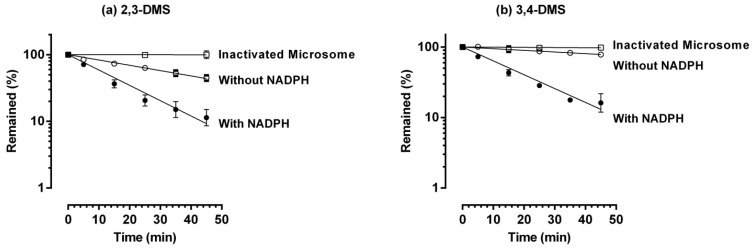
Metabolic stability in liver microsomes. The symbols represent mean values and error bars represent SD. *n* = 6 except *n* = 3 in inactivated microsomes.

The metabolic stabilities of 2,3-DMS and 3,4-DMS were assessed in rat liver microsomes and the results are shown in Figure 3. Both 2,3-DMS and 3,4-DMS displayed good stability in acetonitrile-inactivated microsomes. Similarly, 2,3-DMS and 3,4-DMS had comparable metabolic half-life (2,3-DMS: 14.2 min; 3,4-DMS: 16.6 min) when the microsomal incubation was carried out in the presence of NADPH. In the absence of NADPH, 3,4-DMS was not metabolized much and 78.1% ± 5.3% of the compound still remained after a 45 min microsomal incubation, indicating cytochrome P450 enzymes are the major metabolic enzymes of 3,4-DMS. Interestingly, 2,3-DMS had much more non-cytochrome P450 enzyme mediated metabolism as only 46.6% ± 4.6% of 2,3-DMS remained after incubation in the absence of NADPH. Clearly, the metabolic pathway of these two dimethoxystilbenes appeared to be quite distinct.

### 2.3. Pharmacokinetic Study in Sprague-Dawley Rats

As hydroxypropyl-β-cyclodextrin (HP-β-CD) increases the aqueous solubility of resveratrol and its analogs [8,9,10,11,12], it was used to form water-soluble formulations of 2,3-DMS and 3,4-DMS. The pharmacokinetic profiles were assessed in Sprague-Dawley rats after intravenous or oral administration. To the authors’ knowledge, this is the first report on their pharmacokinetics.

**Figure 4:**
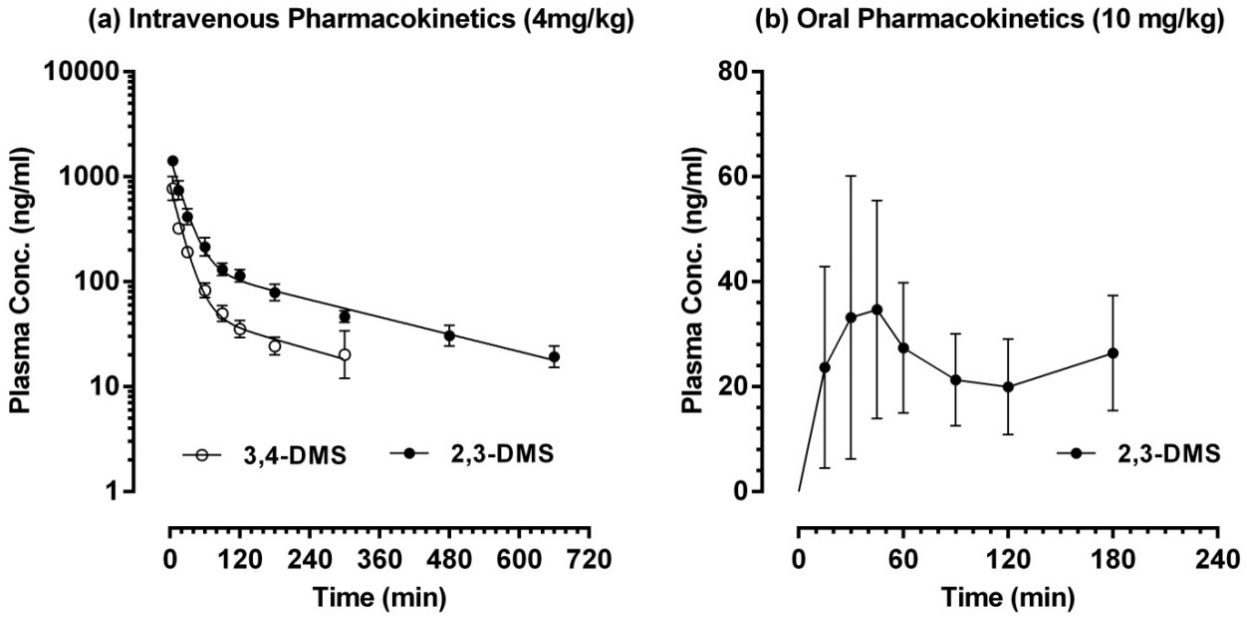
Pharmacokinetic profiles of 2,3-DMS and 3,4-DMS in Sprague-Dawley rats. (**a**) Intravenous administration; (**b**) Oral administration. The symbols represent mean values and error bars represent SD. Intravenous pharmacokinetics: *n* = 5 except *n* = 3 for 3,4-DMS at 300 min; 2,3-DMS oral pharmacokinetics: *n* = 6 at 15 and 30 min; *n* = 4 at 45, 90 and 120 min; *n* = 3 at 60 and 180 min.

The plasma concentration-time profiles of 2,3-DMS and 3,4-DMS are shown in Figure 4a. Upon intravenous injection (4 mg/kg), the concentrations of 2,3-DMS and 3,4-DMS declined following a bi-exponential process, *i.e.*, a distribution phase followed by a terminal elimination phase. Therefore, the classical two-compartment first-order open model was selected to represent the intravenous pharmacokinetic profiles of 2,3-DMS and 3,4-DMS. The plasma concentration *versus* time data of individual rat was fitted into the model and the fitting was excellent (*R^2^* > 0.96), indicating that an appropriate model was chosen. The major pharmacokinetic parameters of 2,3-DMS and 3,4-DMS are listed in Table 4.

**Table 4:** Pharmacokinetic parameters of 2,3-DMS and 3,4-DMS ^a^.

Parameters	2,3-DMS	2,3-DMS	3,4-DMS
Dosing Route	Oral (*n* = 6)	Intravenous (*n* = 5)	Intravenous (*n* = 5)
Dose (mg·kg^−1^)	10	4	4
*A*(ng·mL^−1^)	-	1384.1 ± 251.4	702.7 ± 148.5 **
*B*(ng·mL^−1^)	-	135.6 ± 40.6	55.9 ± 26.1 **
α (10^−2^ × min^−1^)	-	5.02 ± 1.76	5.25 ± 1.45
β (10^−3^ × min^−1^)	-	3.08 ± 0.73	3.85 ± 2.45
*V_c_* (L·kg^−1^)	-	2.71 ± 0.51	5.58 ± 1.73 **
*AUC_0→last_* (10^3^ × min·ng·mL^−1^)	3.86 ± 3.70	69.5 ± 8.5	25.6 ± 7.0 ***
*Cl* (mL·min^−1^·kg^−1^)	-	52.0 ± 7.0	143.4 ± 40.5 ***
*t_1/2 λZ_* (min)	-	288.9 ± 92.9	193.1 ± 123.3
*MTT_0→last_* (min)	83.2 ± 67.4	131.0 ± 4.5	61.4 ± 27.1 ***
*C_max_* (ng·mL^−1^)	37.5 ± 23.7	-	-
*t_max_* (min)	15, 30 or 300	-	-
*F* (%)	2.22 ± 2.13	-	-

^a^ Results were presented as Mean ± SD; ** *p*< 0.01, *** *p* < 0.001 between 2,3-DMS and 3,4-DMS.

Following single bolus intravenous dosing, 2,3-DMS had a medium volume of distribution of the central compartment (*V_c_* = 2.71 ± 0.51 L/kg), quite rapid clearance (*Cl* = 52.0 ± 7.0 mL/min/kg), moderate mean transit time (*MTT_0→last_* = 131.0 ± 4.5 min) but a fairly long terminal elimination half-life (*t_1/2 λZ_* = 288.9 ± 92.9 min). Interestingly, 3,4-DMS displayed a pharmacokinetic profile apparently distinct from 2,3-DMS and it had more extensive distribution (*V_c_* = 5.58 ± 1.7 L/kg), faster clearance (*Cl* = 143.4 ± 40.5 mL/min/kg) and shorter residence (*MTT_0→last_* = 61.4 ± 27.1 min). With a LLOQ of 10 ng/mL, the plasma level of 2,3-DMS all remained quantifiable throughout our sampling period; however, 3,4-DMS dropped to a unquantifiable level in 2 out of 5 rats at 3 h post-administration and it eventually became unmeasurable at 11 h after intravenous dosing. Clearly, 3,4-DMS had much faster elimination than 2,3-DMS.

The intravenous pharmacokinetic profiles of 2,3-DMS and 3,4-DMS show some similarities with those of other complete methoxystilbenes [8,9,10,11,12,13,14]. All of them could be represented by a classical two-compartment first order elimination model. However, their terminal elimination half-life varies. 3,4-DMS had a faster clearance than 2,3-DMS and other methoxystilbenes. Obviously, the location of the methoxy groups had a significant impact on the pharmacokinetics of resveratrol analog. It is of note that a larger apparent volume of distribution also contributes to a faster clearance as clearance is proportional to the apparent volume of distribution.

The oral pharmacokinetic profile of 2,3-DMS was highly erratic (Figure 4b). Only 32 out of 60 post-dosing samples had 2,3-DMS levels higher than our LLOQ (10 ng/mL) and the time to maximal concentration (*t_max_*) varied from 15 min to 5 h. Both the maximal plasma concentration (*C_max_*) and the plasma exposure (*AUC_0→last_*) were very limited, leading to a poor oral bioavailability (*F* = 2.22% ± 2.13%). The oral bioavailability of 3,4-DMS was almost nil as 3,4-DMS could only be quantified in three post-dosing samples collected from one out of six rats. Clearly, the oral bioavailability of 2,3-DMS and 3,4-DMS was problematic.

It is generally believed that the oral bioavailability of a given compound is determined by its aqueous solubility, bio-membrane permeability and metabolic stability. As both 2,3-DMS and 3,4-DMS were given in water-soluble cyclodextrin formulation, the solubility barrier was completed removed. Similarly, these compounds are unlikely to possess poor bio-membrane permeability based on their physicochemical properties, *i.e.*, molecular weight, clogP (Table S1), number of hydrogen bond donor and acceptor, number of rotatable bond, and polar surface area. The poor oral bioavailability of these two dimethoxystilbenes is very likely attributed to the metabolic issue. According to our metabolic stability study, these two stilbenes had a quite short half-life in rat liver microsome. Moreover, the first-pass metabolism in intestine cloud also contribute to the poor oral bioavailability.

Although 2,3-DMS and 3,4-DMS had comparable metabolic half-lives (2,3-DMS: 14.2 min; 3,4-DMS: 16.6 min) in liver microsomes in the presence of NADPH, the measured oral bioavailability of 3,4-DMS was much poorer than 2,3-DMS. This may be due to extra-hepatic metabolism and more extensive tissue distribution of 3,4-DMS. The clearance of 3,4-DMS was found to be three-fold as high as rat hepatic blood flow, indicating extensive extra-hepatic elimination of 3,4-DMS. Besides hepatic metabolism, intestinal first-pass elimination is a common cause of poor oral bioavailability as well. As 3,4-DMS had a larger apparent volume of distribution of the central compartment, it had extensive tissue distribution, leading to a lower plasma level. Therefore, more plasma samples had a 3,4-DMS concentration less than LLOQ and could not be quantified. This could lead to an underestimated oral bioavailability.

Using our HPLC-UV method, we managed to measure 2,3-DMS and 3,4-DMS in rat plasma at a level as low as 10 ng/mL. The quantification sensitivity could be improved if a mass spectrometry detector were applied. In a recent study, RES and its metabolites were quantified with excellent sensitivity using UPLC-MS/MS method with electrospray ionization [15]. However, electrospray ionization mode may not be suitable for completely methoxylated stilbenes and atmospheric-pressure chemical ionization mode may be more appropriate.

In this study, both 2,3-DMS and 3,4-DMS were found to have poor oral bioavailability. Interestingly, some polymethoxystilbenes such as *trans*-3,5,4'-trimethoxystilbene, *trans*-3,5,3',4'-tetramethoxystilbene and *trans*-3,5,3',4',5'-pentamethoxystilbene had excellent or quite good oral bioavailability [8,12,13]. The calculated logP of these compounds are not so different from those of 2,3-DMS and 3,4-DMS to justify the difference in oral bioavailability (Table S1 in Supplementary Material). In addition, the oral bioavailability of pinosylvine (*trans*-3,5-dihydroxystilbene) was much poorer than resveratrol (*trans*-3,5,4'-trihydroxystilbene) [16,17]. Based on such interesting observations, it could postulate an association between poor oral bioavailability and single phenyl ring substitution within the *trans*-stilbene skeleton. However, such hypothesis needs to be explored and confirmed in future studies.

The intravenous pharmacokinetic profiles of 2,3-DMS and 3,4-DMS were more favorable than RES as they had longer elimination half-life, slower clearance and more abundant plasma exposure [16]. However, the oral pharmacokinetic profiles of 2,3-DMS and 3,4-DMS were poorer than RES because their oral bioavailability was even lower than RES [16]. Clearly, although methoxylation is a practical strategy to prevent phase II metabolism, not all complete methylated stilbenes possess an improved pharmacokinetic profile. The location of methoxy groups should be taken into consideration in future design of RES analogs.

## 3. Experimental Section

### 3.1. General Information

White powdery *trans-*stilbene (96% purity) was obtained from Sigma–Aldrich (St. Louis, MO, USA). HPLC/Spectro-grade acetonitrile was purchased from Tedia (Fairfield, OH, USA). 2-Hydroxypropyl-β-cyclodextrin (HP-β-CD) (degree of substitution about 0.6) was from Wacker (Burghausen, Germany). Analytical grade dimethyl sulphoxide (DMSO) was from MP Biomedicals (Solon, OH, USA). Milli-Q water (18.2 MΩ·cm at 25 °C) was generated by a Millipore Direct-Q^®^ ultrapure water system (Billerica, MA, USA) and used to prepare mobile phase and dosing vehicle. Other reagents were at least of analytical grade. Male Sprague-Dawley rat liver microsome (RLM) (BD Gentest, Cat No 452501, Lot 26850) and nicotinamide adenosine dinucleotide phosphate (NADPH) (NADPH Regenerating Solution **A**: BD Gentest, Cat No 451220, Lot 2180641; NADPH Regenerating Solution **B**: BD Gentest, Cat No 451200, Lot 2136637) were procured from BD Biosciences (San Jose, CA, USA). NMR spectra were run on a Varian Unity Inova spectrometer operating at 499.86 (^1^H) and 125.70 MHz (^13^C) and equipped with gradient-enhanced, reverse-detection probe. Chemical shifts (δ) are indirectly referred to tetramethylsilane (TMS) using solvent signals.

### 3.2. Special Precautions

All laboratory procedures involving manipulations of 2,3-DMS, 3,4-DMS and *trans*-stilbene were carried out in dimly lit environment to avoid photo-isomerization.

### 3.3. Synthesis

Oily colourless liquid 2,3-DMS and white powdery 3,4-DMS (both with purity >97%) were synthesized employing a previously published method based on an Arbuzov rearrangement followed by the Horner-Emmons-Wadsworth reactions.

*trans-3,4-Dimethoxystilbene*: Benzyl bromide (3 mL, 25.2 mmol) was stirred with triethyl phosphite (7 mL) under reflux (130 °C) for 5 h. The mixture was diluted with DMF (37 mL) and was stirred with MeONa (2.7238 g, 54.5 mmol) in an ice bath (0 °C) for 30 min. Then 3,4-dimethoxybenzaldehyde (4.7054 g, 28.3 mmol) was added to the reaction flask, and the mixture was stirred at room temperature for 1 h, at 100 °C for 1 h and finally it was kept under stirring overnight at rt. 3,4-DMS (3.9796 g, yield: 66%) was obtained as a pure product by crystallization from methanol of the crude precipitate obtained adding a mixture of methanol-water (1:2) to the reaction mixture. ^1^H-NMR (500 MHz, CDCl_3_, 300 K) δ = 7.51 (2H, d, *J =* 7.8 Hz, H-2', H-6'), 7.36 (2H, bdd, *J =* 7.8, 7.5 Hz, H-3', H-5'), 7.25 (1H, t, *J =* 7.5 Hz, H-4', signal is partially overlapped with residual CHCl_3_), 7.07 (3H, m, H-7, H-2, H-6, overlapped signals), 6.98 (1H, d, *J =* 16.5 Hz, H-8), 6.87 (1H, d, *J =* 7.5 Hz, H-5), 3.96 (3H, s, 3-OCH_3_), 3.91 (3H, s, 4-OCH_3_). ^13^C-NMR (125 MHz, CDCl_3_, 300 K) δ = 149.1 (C, C-3), 148.9 (C, C-4), 137.5 (C, C-1'), 130.5 (C, C-1), 128.6 (CH, C-3', C-5'), 128.4 (CH, C-4'), 127.2 ^#^ (CH, C-7), 126.8 ^#^ (CH, C-8), 126.2 (CH, C-2', C-6'), 119.9 (CH, C-6), 111.2 (CH, C-5), 108.8 (CH, C-2), 55.9 (CH_3_, 3-OCH_3_), 55.8 (CH_3_, 4-OCH_3_). Values with identical superscript (^#^) may be interchanged.

*trans-2,3-Dimethoxystilbene*: Benzyl bromide (3 mL, 25.2 mmol) was dissolved in triethyl phosphite (7 mL) and stirred under reflux at 130 °C for 5 h. The reaction mixture was diluted in DMF (35 mL) and the solution was stirred with MeONa (2.7540 g, 55.1 mmol) at 0 °C for about 30 min. After this time, 2,3-dimethoxybenzaldehyde (4.7071 g, 28.3 mmol) was added to this solution and the mixture was stirred at room temperature for 1 h, and then heated to 100 °C for 1 h. After cooling the reaction was kept under stirring at room temperature overnight. The reaction mixture was quenched with weakly acid solution and extracted with CH_2_Cl_2_ (200 mL × 3). Finally the combined organic layers were washed with water and dried over anhydrous sodium sulphate (Na_2_SO_4_). The recovered organic phase, evaporated to dryness *in vacuo*, was purified by PLC using silica gel (0.063–0.200 mm) and a gradient of diethyl ether in petroleum ether (from 0% to 2%). Purification has returned 3.8709 g of pure 2,3-DMS (yield: 64%). ^1^H-NMR (500 MHz, CDCl_3_, 300 K) δ = 7.64 (2H, d, *J =* 7.5 Hz, H-2', H-6'), 7.59 * (1H, d, *J =* 16.5 Hz, H-7), 7.45 (2H, bdd, *J =* 7.5, 8.0 Hz, H-3', H-5'), 7.35 (m, 2-H, H-4', H-6,overlapped signals), 7.23 * (1H, d, *J =* 16.5 Hz, H-8), 7.14 (1H, bdd, *J =* 8.0, 8.5 Hz, H-5), 6.90 (1H, dd, *J =* 8.0, 1.5 Hz, H-4), 3.96 (3H, s, 3-OCH_3_), 3.93 (3H, s, 2-OCH_3_). Values with identical superscript (*) maybe interchange. ^13^C-NMR (125 MHz, CDCl_3_, 300 K) δ = 152.9 (C, C-3), 146.8 (C, C-2), 137.5 (C, C-1'), 131.3 (C, C-1), 129.7 (CH, C-4'), 128.5 (CH, C-3', C-5'), 127.5 ^#^ (CH, C-7), 126.5 (CH, C-2', C-6'), 123.9 ^#^ (CH, C-8), 122.8 (CH, C-5), 117.7 (CH, C-6), 111.3 (CH, C-4), 60.8 (CH_3_, 2-OCH_3_), 55.9 (CH_3_, 3-OCH_3_). Values with identical superscript (^#^) may be interchanged. NMR spectra are shown in Supplementary Material.

### 3.4. Chromatographic Conditions

All high performance liquid chromatography (HPLC) analyses were carried out with a Shimadzu (Kyoto, Japan) 2010A liquid chromatography system. This HPLC comprised a quaternary gradient low-pressure mixing pump, online degasser, auto-sampler, column oven, dual-wavelength UV–Vis detector and system controller. Class-VP Version 6.12 SP1 (Shimadzu, Kyoto, Japan) was used to control the HPLC and perform data analysis. A reversed-phase HPLC column (Agilent ZORBAX Eclipse Plus C_18_: 250 × 4.6 mm i.d., 5 mm), protected by a guard column (Agilent Zorbax Eclipse Plus C_18_: 12.5 × 4.6 mm i.d., 5 mm), was used to quantify analytes in rat liver microsome and plasma.

The chromatographic conditions were modified from our recent methods for the quantification of various stilbenes [8,9,10,11,12,13,14]. Chromatographic separation was obtained through a 12.5 min gradient delivery of a mixture of acetonitrile and Milli-Q water at flow rate of 1.5 mL/min at 50 °C. Gradient schedule was: (a) 0–4 min, acetonitrile, 51%; (b) 4–5.5 min, acetonitrile, 51%→90%; (c) 5.5–9 min, acetonitrile, 90%; (d) 9–12.5 min, acetonitrile, 51%. UV absorbance of minimum 310 nm and maximum 325 nm were used to detect analytes but only data acquired at 310 nm was used.

### 3.5. Sample Preparation

Stock solutions of analytes were prepared in DMSO to a final concentration of 1 mg/mL, stored at room temperature (24 °C) and protected from light. The internal standard (IS) (*trans-*stilbene) was prepared by dissolving in acetonitrile and diluted to 100 ng/mL (working solution). Equal volumes of rat liver microsome (RLM) and *trans-*stilbene–acetonitrile working solution were mixed to quench the reaction. After vigorous vortexing for 20 s, the samples were centrifuged at 6,000 *g* for for 10 min at 4 °C. Rat plasma samples were processed by adding *trans-*stilbene–acetonitrile working solution to rat plasma in 3:1 ratio. After vigorous vortexing for 20 s, the samples were centrifuged at 10,000 *g* for 4 min at 4 °C. Finally, the supernatant was transferred into a glass insert that was pre-installed in a 1.5 mL auto-sampler vial. During each assay, 75 µL supernatant was injected into the HPLC-UV system.

### 3.6. Method Validation

Validation of the HPLC-UV assay involved assessing linearity, sensitivity, precision, accuracy, precision and stability profiles of 2,3-DMS and 3,4-DMS under various conditions.

Selectivity of elution conditions was examined by comparing chromatograms of blank rat plasma from six different rats and rat plasma samples containing 10 ng/mL of both 2,3-DMS and 3,4-DMS and 100 ng/mL *trans-*stilbene. In subsequent pharmacokinetic experiments, pre-dosing rat plasma samples were collected from all rats (*n* = 22) and selectivity was further confirmed by chromatographic comparison between the pre-dosing and post-dosing rat plasma samples.

Sensitivity was represented by lower limit of quantification (LLOQ). A signal to noise ratio of 5:1 is defined as LLOQ.

The ratio between peak area of 2,3-DMS and *trans*-stilbene (internal standard) was adopted as analytical response. Linear regression was analyzed using GraphPad Prism 5, version 5.03 for Windows (GraphPad Software, San Diego, CA, USA) via least sum-of-squares method, where *x* was concentration of 2,3-DMS, *y* was analytical response and a weighting factor of 1/*x^2^* was used. Calibration standards of concentrations 10, 50, 100, 250, 500, 1000, and 1500 ng/mL were measured on 5 consecutive days to access linearity. Five replicates of calibration standards were analyzed on Day 3 while duplicates of calibration standards were analyzed on other days to determine intra- and inter-day assay respectively. Similarly, quality control (QC) samples at 25, 400, 1400 ng/mL were used for assessing intra- and inter-day precision and accuracy. Precision was defined by coefficient of variation while accuracy was determined by percentage of measured concentration against the corresponding nominal concentrations. The precision and accuracy of 3,4-DMS was assessed in the same way.

Stabilities of 2,3-DMS in stock solution were evaluated after storage at room temperature for eight days. Stability of 2,3-DMS in rat plasma under different conditions was profiled with QC samples. Freeze-thaw stability was assessed after three freeze (−80 °C)–thaw (24 °C) cycles. Short-term room temperature stability (24 °C) of 2,3-DMS in rat plasma was examined after storing such samples at room temperature for 6 h. Long-term storage stability of 2,3-DMS in rat plasma was studied after being stored at −80 °C for 10 days. Post-preparative stability study was conducted by re-analyzing samples (kept in auto-sampler vial at room temperature) 24 h later. The stability of 3,4-DMS was assessed in the same way. Samples were considered stable if analytical responses were within 100% ± 15% of corresponding freshly prepared samples.

### 3.7. Rat Liver Microsome Metabolic Stability Investigation

RLM metabolic stabilities of 2,3-DMS and 3,4-DMS were examined by mixing Sprague-Dawley RLM with potassium phosphate buffer (pH = 7.4), NADPH regenerating solution **B** and either compounds to achieve a final compound concentration of 0.6 µg/mL. Negative control was set up, using Milli-Q water in place of NADPH. Chemical stability of compound in inactivated RLM was checked using similar system with acetonitrile/Milli-Q mixture instead of phosphate buffe.

Pre-incubation was done in 37 °C water bath for 5 min before NADPH **A** was added to initiate reaction. Aliquots were drawn at 0, 5, 15, 25, 35 and 45 min and quenched using ice cold *trans-*stilbene–acetonitrile working solution. Chemical stability was monitored at 0, 25 and 45 min. The samples were further processed for HPLC analysis as described above.

To assess extent of metabolism, percentage of compound remaining at each time point with respect to 0 min was calculated. Mean and standard deviation was obtained from column statistics using GraphPad Prism 5, version 5.03 for Windows, via raw means from the six replicates of each setup. Elimination half-lives were obtained from non-linear regression via one-phase exponential decay, where *x* was time in minutes, *y* was percentage remaining with respect to 0 min and a weighting factor of 1/*y^2^* was used.

### 3.8. Pharmacokinetic Profiling in Male Sprague-Dawley Rats

This animal model has been used to investigate the pharmacokinetics of various stilbenes [8,9,10,11,12,13,14]. The animal handling protocol was reviewed and approved by the Institutional Animal Care and Use Committee of the National University of Singapore (NUS). All animal experiments were carried out in Comparative Medicine Centre of NUS. Sprague-Dawley rats (male, 346 ± 18 g, bred by Centre for Animal Resources, NUS) were maintained on a 12 h light/dark cycle with free access to food and water. Polyethylene tube (inner diameter (I.D.) 0.58 mm, outside diameter (O.D.) 0.965 mm, Becton Dickinson, Sparks, MD, USA) was implanted into the right jugular vein under anaesthesia on the day before pharmacokinetic study. This catheter was used for intravenous drug administration as well as for blood sampling.

Twenty-two rats were divided into four groups. Group 1 and Group 2 (*n* = 5) received single intravenous bolus dose (4 mg/kg) of 2,3-DMS and 3,4-DMS respectively. Serial blood samples were collected before dosing (0 min) and at 5, 15, 30, 60, 90, 120, 180, 300, 480 and 660 min post-dosing. Group 3 and Group 4 (*n* = 6) received single oral dose (10 mg/kg) of 2,3-DMS and 3,4-DMS respectively through oral gavage and serial blood samples were collected before dosing (0 min) and at 15, 30, 45, 60, 90, 120, 180, 300, 480 and 660 min post-dosing. 0.3 mL heparin-saline (10 I.U./mL) was used to flush the cannular after each intravenous injection or blood sampling to maintain its patency. Blood samples were centrifuged at 3500 *g* at 4 °C for 5 min and plasma was collected and stored at −80 °C.

Pharmacokinetic parameters were derived using WinNonlin standard version 1.0 (Scientific Consulting Inc., Apex, NC, USA). Intravenous pharmacokinetic profiles of both compounds displayed typical bi-exponential decline, thus their plasma concentration-time data were fitted into classical two-compartment first-order open model (*C* = *A·e^−α·t^* + *B·e^−β·t^*) using a weighting factor of 1/*y^2^* [13,17]. Plasma exposure (area under the plasma concentration-time curve from 0 min to last measurable point (*AUC_0→last_*)) in rats receiving intravenous administration was calculated by the linear/log trapezoidal rule while the linear trapezoidal rule was used to calculate the plasma exposure in rats receiving oral administration [13,17]. Clearance (*Cl_0→last_*), mean transit time (*MTT_0→last_*) and terminal half-life (*t_1/2 λZ_*) were calculated using non-compartmental analysis. Maximal plasma concentration (*C_max_*) and the time required to reach (*t_max_*) were from direct experimental observations. Absolute oral bioavailability (*F*) of 2,3-DMS was calculated through reported method [13,17].

### 3.9. Statistical Analysis

All statistical calculations were calculated using GraphPad InStat, Version 3.10, 32 bit for Windows. Two-tailed unpaired t-test was used to compare intravenous pharmacokinetic parameters of both compounds. Two-tailed Chi square (χ^2^) test and Fisher’s exact test were used to compare the contingency tables for number of quantifiable intravenous plasma samples and number of quantifiable oral plasma samples of both compounds respectively. For all analysis, probability level of *p* ≤ 0.05 represented criterion of statistical significance.

## 4. Conclusions

A simple and sensitive HPLC-UV method was developed and validated for the quantification of 2,3-DMS and 3,4-DMS in rat plasma. The pharmacokinetic profiles of 2,3-DMS and 3,4-DMS were assessed in Sprague-Dawley rats. The location of the methoxy groups was found to have a significant impact on the pharmacokinetics of the resveratrol analogs. The current study also suggested that single phenyl ring substitution could be associated with poor oral bioavailability and metabolic instability. The information obtained in this study will benefit the appropriate design of resveratrol derivatives in future.

## Supplementary Materials

Supplementary materials can be accessed at: http://www.mdpi.com/1420-3049/19/7/9577/s1.
