# Supplementary material for: Quantification of the Resveratrol Analogs trans-2,3-Dimethoxy-stilbene and trans-3,4-Dimethoxystilbene in Rat Plasma: Application to Pre-Clinical Pharmacokinetic Studies
Source: Molecules. 2014 Jul 7;19(7):9577–90. doi: 10.3390/molecules19079577 (PMC6271088; doi:10.3390/molecules19079577)
Supplement: Supplementary file 1 [file molecules-19-09577-s001.pdf]

Supplementary Material

Figure S1. ^{1}H-NMR spectrum ($CDCl_3$, 500 MHz) of 3,4-DMS.

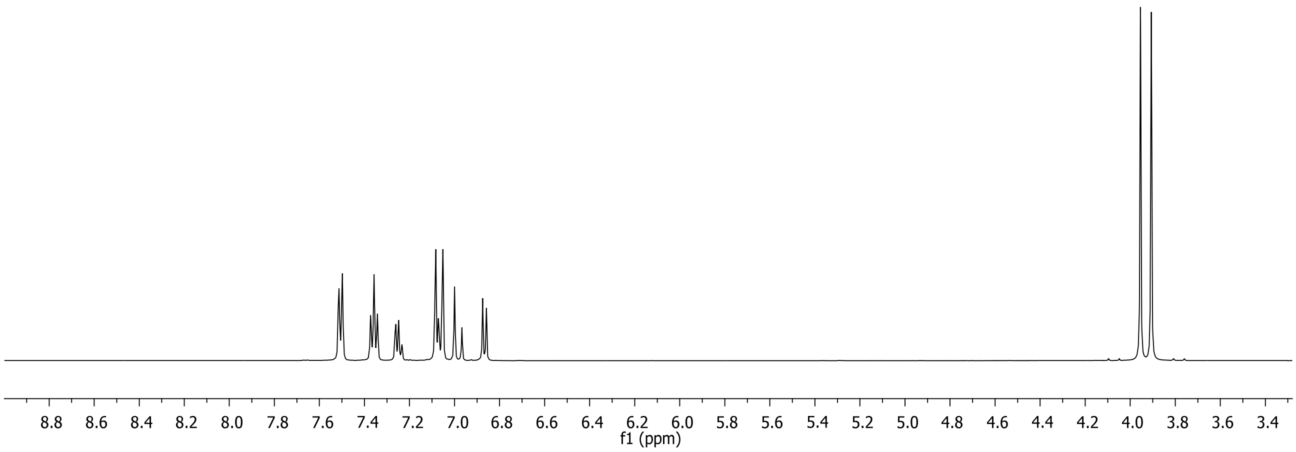

Figure S2. ^{13}C-NMR spectrum ($CDCl_3$, 125 MHz) of 3,4-DMS.

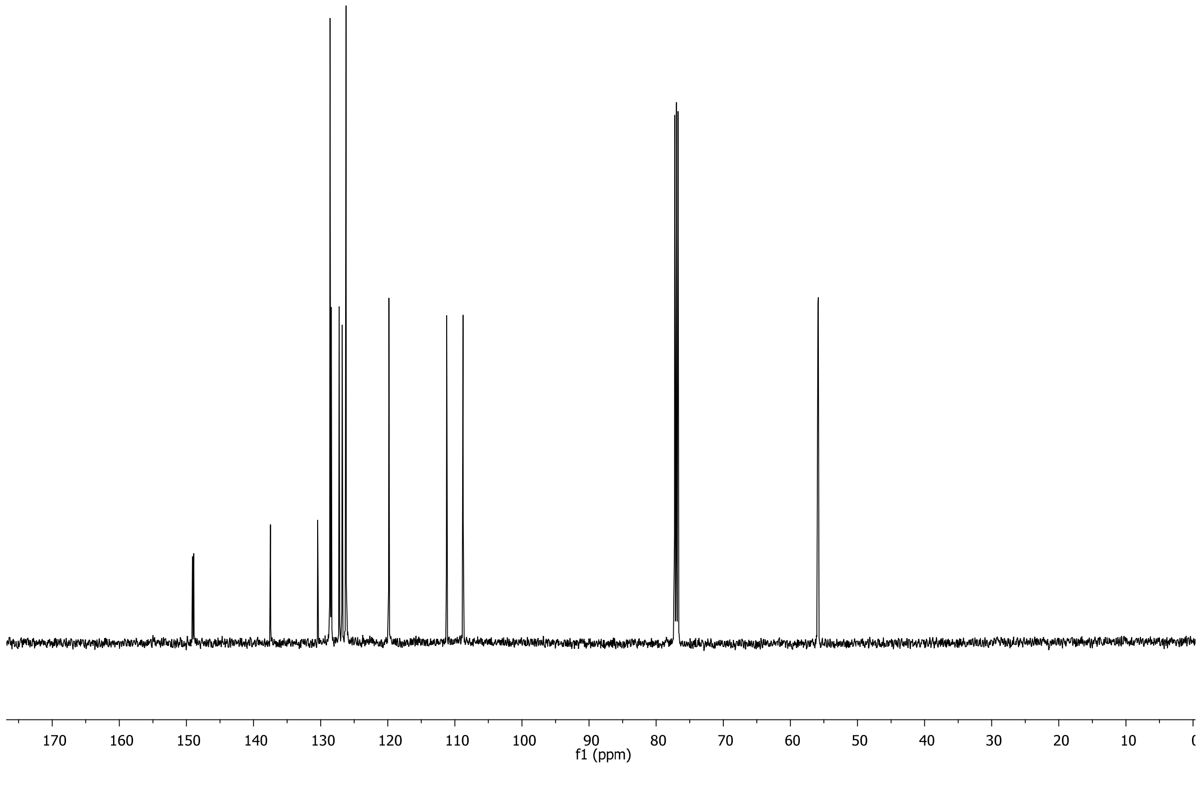

Figure S3. ^{1}H-NMR spectrum ($CDCl_3$, 500 MHz) of 2,3-DMS.

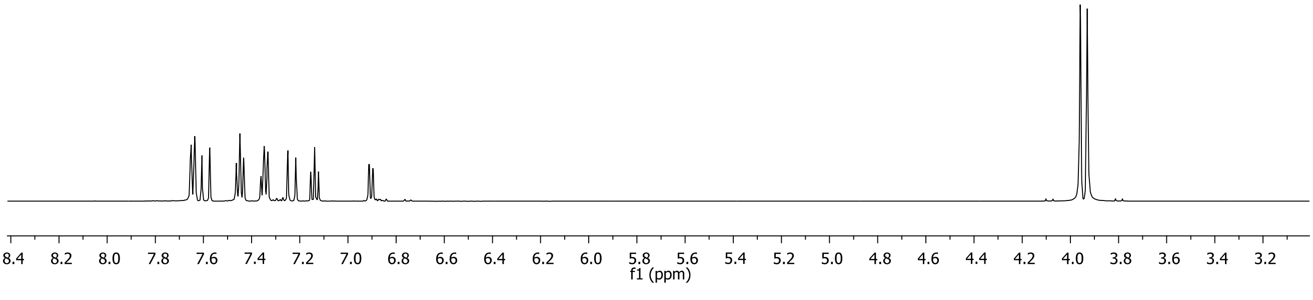

Figure S4. ^{13}C-NMR spectrum ($CDCl_3$, 125 MHz) of 2,3-DMS.

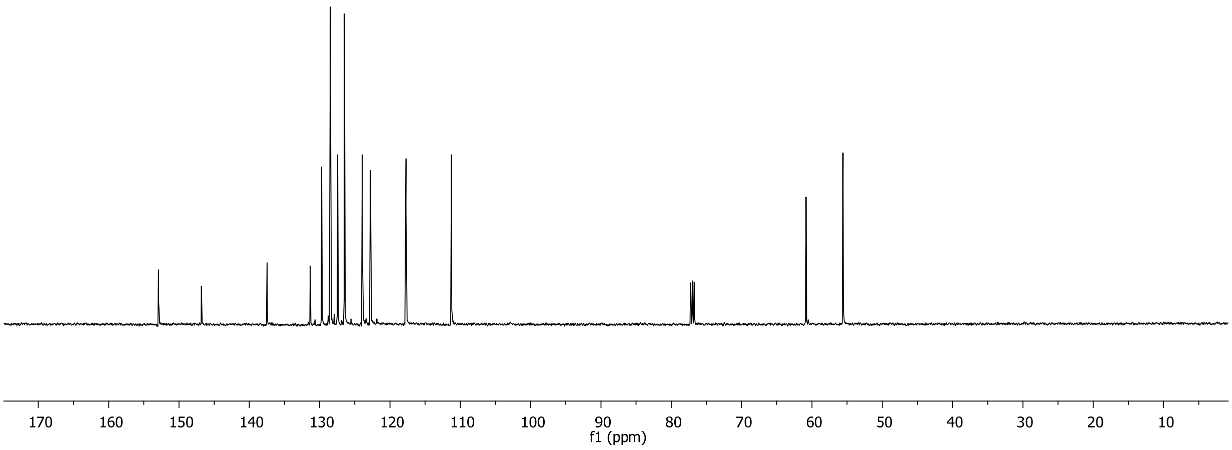

Table S1. Calculated LogP of polymethoxystilbenes.

Name	Formula	Log P
3,5,4'-trimethoxysilbene	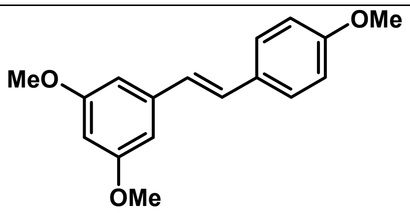	4.32 ± 0.26
3,4,3',4'-tetramethoxystilbene	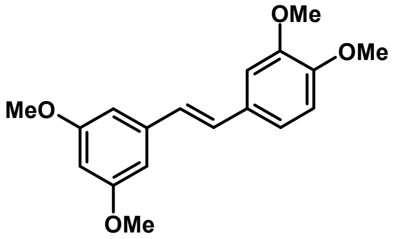	3.98 ± 0.35
2,3-dimethoxystilbene	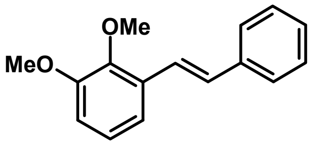	3.94 ± 0.24
stilbene	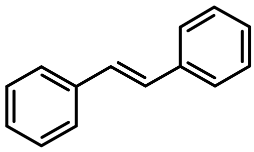	3.89 ± 0.19
3,4,5,2',4'-pentamethoxystilbene	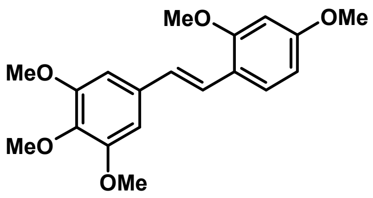	3.87 ± 0.36
3,4,5,3',5'-pentamethoxystilbene	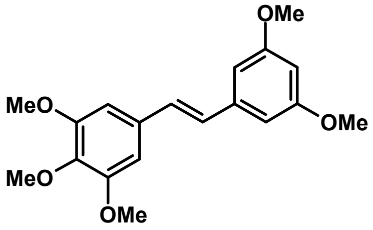	3.72 ± 0.36

Table 1S. *Cont.*

Name	Formula	Log P
3,4-dimethoxystilbene	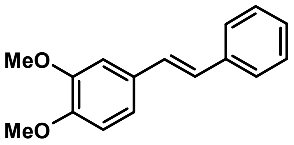	3.61 ± 0.24
3,4,5,4'-tetramethoxystilbene	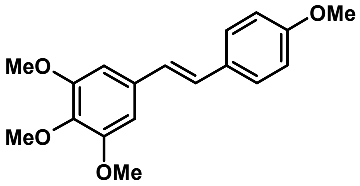	3.40 ± 0.35
